# Veratridine produces distinct calcium response profiles in mouse Dorsal Root Ganglia neurons

**DOI:** 10.1038/srep45221

**Published:** 2017-03-24

**Authors:** Zainab A. Mohammed, Ciara Doran, David Grundy, Mohammed A. Nassar

**Affiliations:** 1 Biomedical Science, University of Sheffield, Sheffield S102TN, UK

## Abstract

Nociceptors are a subpopulation of dorsal root ganglia (DRG) neurons that detect noxious stimuli and signal pain. Veratridine (VTD) is a voltage-gated sodium channel (VGSC) modifier that is used as an “agonist” in functional screens for VGSC blockers. However, there is very little information on VTD response profiles in DRG neurons and how they relate to neuronal subtypes. Here we characterised VTD-induced calcium responses in cultured mouse DRG neurons. Our data shows that the heterogeneity of VTD responses reflects distinct subpopulations of sensory neurons. About 70% of DRG neurons respond to 30–100 μM VTD. We classified VTD responses into four profiles based upon their response shape. VTD response profiles differed in their frequency of occurrence and correlated with neuronal size. Furthermore, VTD response profiles correlated with responses to the algesic markers capsaicin, AITC and α, β-methylene ATP. Since VTD response profiles integrate the action of several classes of ion channels and exchangers, they could act as functional “reporters” for the constellation of ion channels/exchangers expressed in each sensory neuron. Therefore our findings are relevant to studies and screens using VTD to activate DRG neurons.

Dorsal Root Ganglia (DRG) contain a heterogeneous population of sensory neurons that detect noxious and innocuous stimuli. DRG neurons that respond to noxious stimuli are known as nociceptors. Inflammation and nerve injury sensitise sensory neurons and result in hyperalgesia and allodynia^1^,^2^. Understanding the physiology and pathophysiology of nociceptors is important for the development of analgesic drugs. Nociceptors express multiple subtypes of voltage-gated sodium channels (VGSCs) which are a key determinant of their excitability. Inflammation and injury-induced sensitisation of nociceptors is in part mediated through changes in expression and/or properties of VGSCs^1^,^2^,^3^.

Drug screens for VGSC blockers use functional assays to test their sensitivity. These include FLIPR, calcium imaging and patch clamp electrophysiology; and employ Veratridine (VTD) as a VGSC “agonist” to test the pharmacological properties of candidate VGSC ligands^4^. VTD is a natural, lipid-soluble alkaloid from the Liliaceae family that binds to the S6 in Domain I and IV of VGSCs; a site-2 neurotoxin^4^,^5^,^6^. VTD binds to open VGSCs and prevents the channels from entering into the inactivated state^7^,^8^,^9^. VTD acts predominantly on tetrodotoxin-sensitive (TTX-S) VGSCs^10^. Despite its wide use there is little information on how VTD responses relate to the different subpopulations of DRG neurons; particularly nociceptors. Given the functional and molecular heterogeneity of DRG neurons we hypothesised that VTD produces heterogeneous responses in primary sensory neurons. A better characterisation of the VTD responses in DRG neurons may provide a novel functional classification of sensory neurons.

## Results

### Veratridine produces TTX-sensitive responses

**Figure 1:**
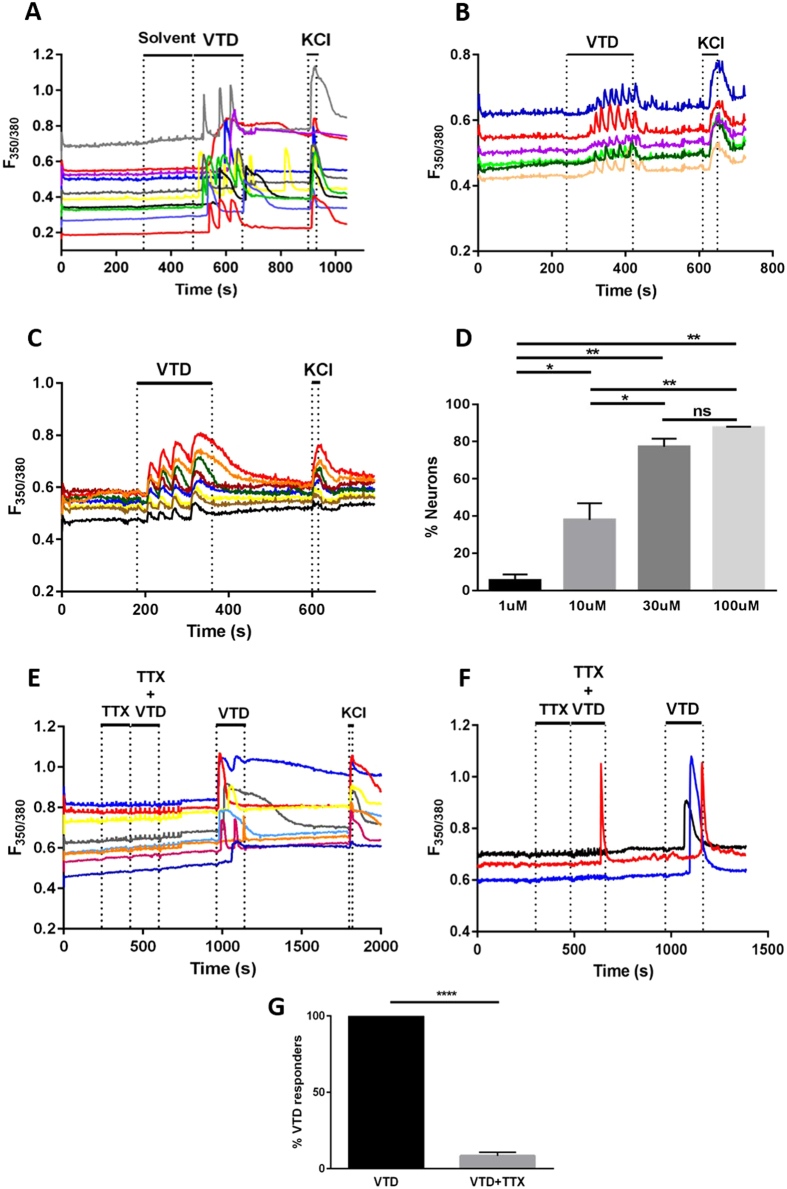
Veratridine elicits TTX-sensitive calcium responses in DRG neurons. (**A**) Example traces of ratiometric (F350/380) increases in Fura-2 fluorescence following application of 30 μM veratridine (VTD) and 40 mM KCl to cultured DRG neurons, Nueroblastoma-2a (N2a) cells **(B)** and SH-5Y5 cells **(C)**. Each trace represents the response of a single cell. VTD but not solvent (ethanol) elicited responses of heterogeneous profiles in DRG neurons but not in any of the cell lines. (**D**) Percentages of neurons activated by a range of VTD concentrations from potassium responsive neurons. Response rates are 5.4 ± 3 for 1 μM (20/303 cells), 37.8 ± 9 for 10 μM (131/325 cells), 77.2 ± 4 for 30 μM (119/151 cells) and 87.5 ± 0.3 for 100 μM (272/311 cells); from N = 2 mice. One-way analysis of variance with Tukey’s post-test, *P < 0.05, **P < 0.01 and ***P < 0.001. (**E**) Example traces of response to 30 μM VTD in presence and then absence of 0.3 μM Tetrodotoxin (TTX). (**F**) An example trace of a neuron responding to VTD in the presence of 0.3 μM TTX. (**G**) TTX blocked 92% of VTD responses. Only 8 ± 2 (10/98 cells) of neurons responded to VTD in the presence of TTX. Data shown are mean ± SEM, Two-tailed paired Student’s t-test, ****P < 0.0001.

VTD elicited heterogeneous calcium responses that differed in their profile and onset in sensory neurons, Fig. 1A. This is in contrast to the homogenous response profile elicited in the neuroblastoma cell lines N2a^11^ and SH-SY5Y^12^, which are commonly used mouse and human neuronal cell lines (respectively), Fig. 1B,C. Different concentrations of VTD ranging from 15 to 200 μM have been used to activate VGSCs in a variety of cell types^7^,^10^,^13^,^14^,^15^. We therefore examined a wide range of VTD concentrations (0.001, 0.1, 1, 10, 30, and 100 μM) on cultured mouse DRG neurons. Since 200 μM VTD has been shown to inhibit voltage-gated potassium channels (VGKCs)^13^ we did not use concentrations greater than 100 μM. There was a concentration dependent increase in the number of responding neurons from a threshold of 1 μM to which 5% of neurons responded. 10 μM elicited a response in 38% of neurons while 30 μM and 100 μM VTD elicited responses in 77% and 88% of neurons, respectively, Fig. 1D. Subsequent experiments were performed using 30 μM VTD because of the robust response at this concentration and to minimise potential effects on VGKCs. Responses to 30 μM VTD varied in latency from 30 to 150 seconds as shown in the example traces in Fig. 1A. Therefore, we standardised the period of VTD application to three minutes. Pre-treatment of neurons with TTX (0.3 μM) blocked VTD responses in 92% of VTD-responsive neurons, Fig. 1E–G. Of note, VTD responses elicited in the presence of TTX had a response profile characterised by a single peak with a delayed onset (towards the end of the 3 minutes of VTD application period), Fig. 1F.

### Veratridine produces four distinct calcium response profiles that occur at different frequencies

**Figure 2:**
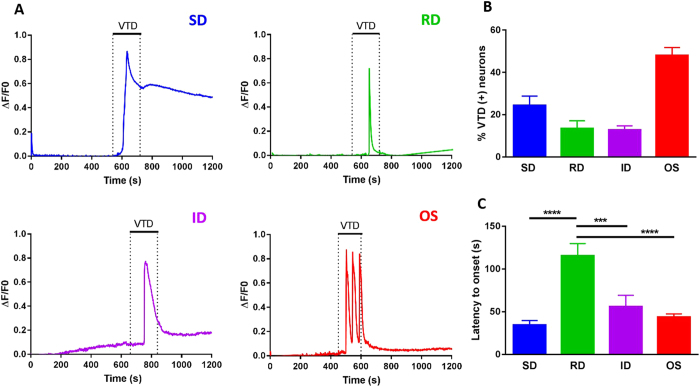
Veratridine produces four calcium response profiles that differ in their frequency of occurrence and latency to peak. (**A**) Representative traces of the four VTD response profiles observed in cultured DRG neurons. SD (slow decay) in blue has the slowest decay rate of calcium signal, followed by ID (intermediate decay) in purple and then RD (rapid decay) in green. Responses with two or more peaks are called oscillatory (OS) in red. (**B**) Frequency of occurrence of the four VTD response profiles in VTD-responsive neurons (N = 8 mice, 494 cells). The OS profile is the most frequent (48 ± 4%), followed by the SD profile (24.4 ± 4%). The RD (13.4 ± 4%) and ID (12.7 ± 2%) profiles have similar frequencies. (**C**) Mean response latency for the four VTD response profiles (N = 3 mice, 143 cells). Only neurons that have the same onset to KCl were included in this analysis to exclude differences due to rate of perfusion. The RD (115.4 ± 15 s) profile has the longest latency while the SD (34.2 ± 5 s), ID (55.8 ± 13 s) and OS (43.5 ± 4 s) profiles have similar latencies. Data shown are mean ± SEM. One-way analysis of variance with Tukey’s post-test, ***P < 0.001 and ****P < 0.0001.

We classified VTD responses into four distinct profiles, Fig. 2A. Three profiles were characterised by a single peak with a rapid rise in intracellular calcium [Ca]i levels but differed in their decay rate. The Slow Decay (SD) profile returned to baseline over 15–40 min following VTD washout, Fig. 2A (blue). In the Intermediate Decay (ID) profile, calcium responses returned to baseline within 2–5 min, Fig. 2A (purple). The Rapid Decay (RD) profile was characterised by a transient [Ca]i peak which returned to baseline during the application period, Fig. 2A (green). The fourth profile was characterised by a multi-peak response which we thus called the Oscillatory profile (OS), Fig. 2A (red). In some OS neurons the response ended prior to the end of VTD application while in others it continued to oscillate during the wash. The four VTD response profiles varied in their frequency of occurrence. The OS profile was the most frequent accounting for 48% of all VTD-responsive neurons, followed by the SD profile (24%) and equal frequencies for the ID and RD profiles (13% each), Fig. 2B. Furthermore, the four profiles differed in their onset, calculated as the latency to peak from the start of VTD application. The RD profile had the longest latency which was twice that of the other three profiles, Fig. 2C. The onset difference was inherent to neurons and not due to variation in the rate of perfusion since all neurons responded with the same latency to high potassium. A very small number of VTD-responsive neurons had a “ramp” like profile characterised by a very slow rise and decay of [Ca]i levels (not shown). These neurons were classified as “undefined” in further experiments and were not included in our characterisation because they were too infrequent (less than 1%) to include in our analysis and draw any conclusions about.

### The Veratridine SD response profile is enriched in large diameter neurons

**Figure 3:**
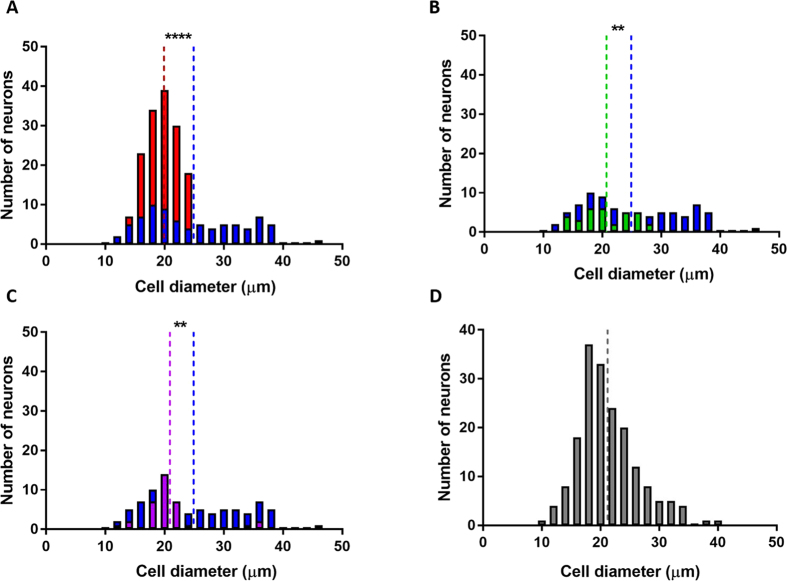
Veratridine OS, RD and ID response profiles occur in neurons smaller than 30 μm in diameter. (**A**) Histogram of soma diameter distribution of neurons with the SD profile in blue (mean 42.9 ± 1, median = 23.1 μm; 79 cell) with superimposed distribution of neurons with the OS profile in red (mean 19.9 ± 0.3 μm, median = 19.7 μm; 161 cell). (**B**) Histogram of soma diameter of neurons with the SD profile versus neurons with the RD profile in green (mean 20.7 ± 0.8 μm, median = 20.1 μm; 33 cell). (**C**) Histogram of soma diameter of neurons with the SD profile versus neurons with the ID profile in purple (mean 20.9 ± 0.8 μm, median = 20.1 μm; 35 cell). (**D**) Histogram of soma diameter of VTD-unresponsive neurons (mean 21.2 ± 0.4 μm, median = 20.4 μm; 181 cell). Histograms are in 2 μm bins. Vertical dotted line indicates the mean value for each distribution. All diameter measurements were taken from N = 8 mice and compared by Two-tailed paired Student’s t-test, *P < 0.05 and ****P < 0.0001.

DRG neurons are classified according to their soma size into small, medium and large diameter neurons^16^,^17^,^18^. Soma size of DRG neurons correlates with functional modalities. Small and medium sized neurons tend to be nociceptors and innocuous thermoreceptors, while large diameter neurons are more likely to be proprioceptors and low threshold mechanoreceptors^19^,^20^. Neuronal soma size in our DRG cultures ranged from 10 to 45 μm. Analysis of soma size and VTD response profiles showed that although the SD profile occurs in all neuronal sizes (mean 25 μm), it was the most prevalent profile in neurons larger than 30 μm in diameter (82% of these neurons had the SD profile). Neurons with the RD, OS and ID profiles had the narrowest size range with almost all smaller than 30 μm in diameter (100, 99 and 91%, respectively). The mean soma diameter of neurons with the RD, OS and ID VTD response profiles was smaller than that of SD neurons (RD 21 μm, OS 20 μm and ID 21 μm), Fig. 3A–C. Of note, the majority (94%) of VTD-unresponsive neurons were smaller than 30 μm (mean 21 μm), Fig. 3D.

### The Veratridine SD and RD response profiles are under-represented in capsaicin sensitive neurons

**Figure 4:**
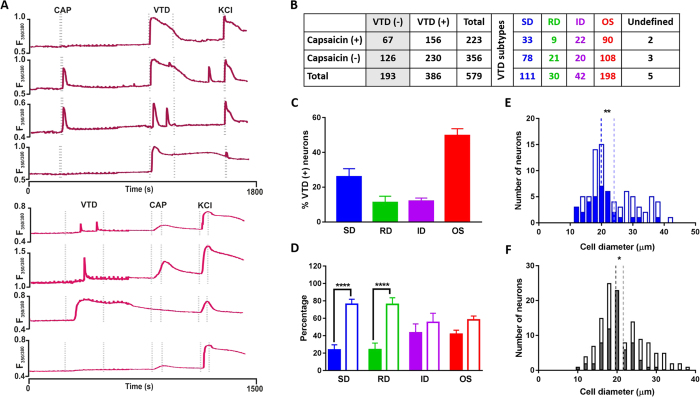
Veratridine SD and RD response profiles are under-represented in capsaicin-sensitive neurons. (**A**) Example traces from the two drug application protocols used. Top four traces from experiments where VTD was applied after 200 nM capsaicin. Bottom traces from experiments where VTD was applied before 1 μM capsaicin. In the former case, a lower capsaicin concentration was used to avoid potential indirect suppression of VGSC by capsaicin^42^ (**B**). Capsaicin sensitivity and VTD response profiles of 579 neurons from N = 8 mice (combined from the two protocols in A). (**C**) The frequency of occurrence of the four VTD response profiles in the 386 VTD-responsive neurons shows the same order as in Fig. 2B. The OS profile is the most frequent (49.7 ± 4%) then the SD (26 ± 5%) followed by the ID (12 ± 2%) and the RD (11.1 ± 4%). (**D**) The proportion of capsaicin-sensitive neurons (closed bars) and capsaicin-insensitive neurons (open bars) within the four VTD response profiles. A larger proportion of SD (76.2 vs. 23.8 ± 6%) and RD neurons (76 vs. 24 ± 8%) is insensitive to capsaicin. OS (58.1 vs. 41.9 ± 5%) and ID neurons (55.3 vs. 43.4 ± 10%) show no difference. Frequency was from total number of neurons in each profile (n_(SD)_ = 111, n_(RD)_ = 30, n_(ID)_ = 42, and n_(OS)_ = 198 neurons). Data shown are mean ± SEM. One-way analysis of variance with Sidak’s post-test, ****P < 0.0001. (**E**) Diameter of SD neurons sensitive to capsaicin is smaller (closed bars, mean 19.9 ± 0.9 μm, median 19.3 μm, n = 33 cell) than diameter of capsaicin insensitive neurons (open bars; 24 ± 0.8 μm, median 21 μm, n = 78 cell). (**F**) Diameter of VTD-unresponsive neurons showing capsaicin-sensitive (closed bars, mean 19.7 ± 0.4 μm, median 19.8 μm, n = 67 cell) and capsaicin-insensitive populations (open bars, mean 21.6 ± 0.5 μm, median 20.3 μm, n = 126 cell). In (**E**,**F**), dotted lines represent the mean. Two-tailed unpaired Student’s t-test, *P < 0.05 and **P < 0.01.

We next investigated how the four VTD response profiles correlate with a commonly used functional marker of nociceptors. Capsaicin is a TRPV1 agonist used to activate peptidergic nociceptors^21^. In order to examine the relationship between VTD response profiles and sensitivity to capsaicin we applied them sequentially in a single calcium imaging protocol as illustrated in Fig. 4A. The order in which the two agonists were applied did not influence the proportion of responding neurons nor the frequency of occurrence of VTD response profiles, Supplementary Fig. S1. Therefore, we combined results from both experiments in subsequent analysis, Fig. 4B. The frequency of occurrence of the four response profiles in these neurons was in the same order as obtained without capsaicin, Fig. 4C. The percentage of capsaicin sensitive neurons in our cultures (32%) is comparable to published reports^22^. Capsaicin sensitive neurons were approximately twice as likely to respond to VTD (156 of 223, 70%) than not (67 of 223, 30%), Fig. 4B. We next examined VTD-responsive neurons for the relationship between each of the four VTD response profiles and capsaicin sensitivity. The proportions of capsaicin-sensitive and insensitive neurons within the OS and ID profiles were not significantly different. However, there was a clear bias in neurons with the SD and RD profiles towards capsaicin insensitivity, Fig. 4D.

As the SD profile occurred in a wide range of neuronal sizes, we examined the relationship between neuronal size and capsaicin sensitivity in the 111 neurons with the SD profile. Capsaicin-sensitive SD neurons had a smaller mean diameter than capsaicin-insensitive SD neurons, Fig. 4E, which is consistent with the known tendency of capsaicin sensitive neurons to be small to medium in diameter. Interestingly, capsaicin activated 35% (67 of 193) of VTD-unresponsive neurons which were all smaller than 30 μm, Fig. 4F. From all the above, the SD and RD profiles are enriched in capsaicin-insensitive neurons.

### The Veratridine SD and RD response profiles are under-represented in α, β-methylene ATP sensitive neurons

**Figure 5:**
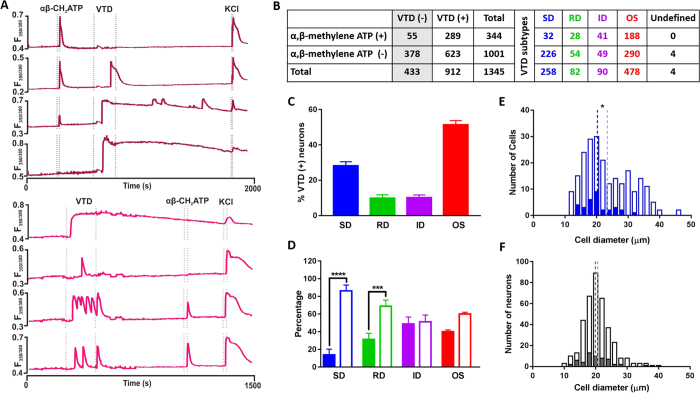
Veratridine SD and RD response profiles are under-represented in α, β-methylene ATP- sensitive neurons. (**A**) Example traces from the two drug application protocols used. (**B**) α, β-methylene ATP sensitivity and types of VTD response profile of the 1345 neurons sampled from N = 9 mice (combined from the two protocols in A). (**C**) The frequency of occurrence of VTD profiles in the 912 VTD-responsive neurons shows the same order as in Fig. 2B. The OS profile is the most frequent (51.4 ± 2%) then SD (28.1 ± 2%) followed by ID (10.1 ± 2%) and RD (9.8 ± 2%). (**D**) The proportion of α, β-methylene ATP-sensitive neurons (closed bars) and α, β-methylene ATP-insensitive neurons (open bars) within each of the four VTD response profiles. A larger proportion of SD (86.3 vs. 13.7 ± 6.6%) and RD neurons (68.9 vs. 31.1 ± 7) is insensitive to α, β-methylene ATP. Neurons with the OS (60 vs. 40 ± 2%) and ID (51.1 vs. 48.9 ± 8%) profile show no difference in the proportion of α, β-methylene ATP-sensitive and -insensitive neurons. Frequency was calculated from total number of neurons in each profile (n_(SD)_ = 258, n_(RD)_ = 82, n_(ID)_ = 90, and n_(OS)_ = 478 neurons). Data shown are mean ± SEM. One-way analysis of variance with Sidak’s post-test, ***P < 0.001 and ****P < 0.0001. (**E**) Histogram of diameter of neurons with the SD response profile showing that α, β-methylene ATP-sensitive neurons (closed bars, mean 20.2 ± 0.8 μm, median 19.9 μm, n = 32 cell) are significantly smaller than α, β-methylene ATP-insensitive neurons (open bars; mean 23.4 ± 0.5 μm, median 21.3 μm, n = 226 cell). (**F**) Histogram of diameter of neurons unresponsive to VTD showing α, β-methylene ATP-sensitive population (closed bars, mean 19.9 ± 0.6 μm, median 19.2 μm, n = 55 cell) and α, β-methylene ATP -insensitive population (open bars, mean 20.6 ± 0.2 μm, median 20.2 μm, n = 378 cell). In (**E**,**F**), dotted lines represent the mean. Two-tailed unpaired Student’s t-test, *P < 0.05 and **P < 0.01.

We next investigated how the four VTD response profiles correlate with sensitivity to another commonly used marker for nociceptors. α, β-methylene ATP is a specific agonist for P2×3 receptors that marks the non-peptidergic population of nociceptors^23^,^24^,^25^,^26^. A preliminary experiment showed that responses to 1 μM α, β-methylene ATP (the EC50 for P2×3) can be completely inhibited by TNP-ATP, a selective P2×3 antagonist^27^ (data not shown). Therefore, we applied 1 μM α, β-methylene ATP with VTD to examine the relationship between the four VTD response profiles and sensitivity to α, β-methylene ATP. We applied the two agonists in alternate order, Fig 5A. As with capsaicin, the order in which VTD and α, β-methylene ATP were applied did not significantly influence the proportion of responding neurons nor the frequency of occurrence of VTD profiles, Supplementary Fig. S2. The frequency of occurrence of the four VTD response profiles was as obtained previously, Fig. 5C. The percentage of α, β-methylene ATP-responsive neurons (26%) was comparable to percentage of P2×3 positive neurons reported in immunolabelling studies^23^,^26^. α, β-methylene ATP-responsive neurons are approximately five times more likely to respond to VTD (289/344, 84%) than not (55/344, 16%), Fig. 5B. We next examined the 912 VTD-responsive neurons for a relationship between each of the four VTD response profiles and α, β-methylene ATP sensitivity. The proportions of α, β-methylene ATP-sensitive and insensitive neurons within the OS and ID profiles were not significantly different, Fig. 5D. However, like with capsaicin, there was a clear bias in neurons with the SD and RD profiles towards α, β-methylene ATP insensitivity.

The SD subpopulation of neurons sensitive to α, β-methylene ATP had a smaller mean size (20 μm) compared to the subpopulation insensitive to α, β-methylene ATP (23 μm), Fig. 5E. Interestingly, 98% of VTD-unresponsive but α, β-methylene ATP-sensitive neurons were less than 30 μm in diameter, Fig. 5F. From all the above, the SD and RD profiles are enriched in α, β-methylene ATP-insensitive neurons.

### The Veratridine OS, ID and RD response profiles are enriched in nociceptors

**Figure 6:**
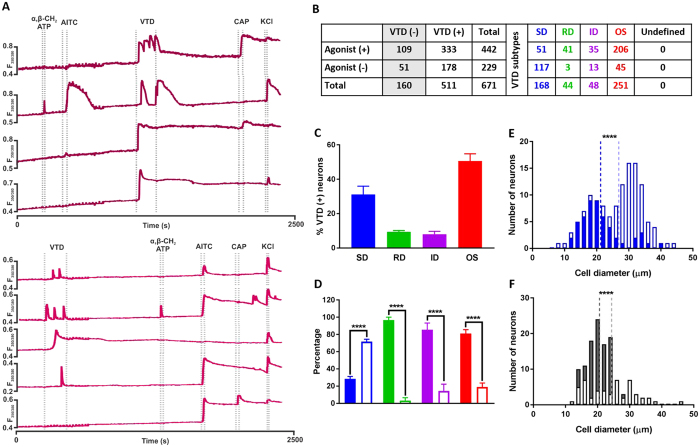
The Veratridine OS, ID and RD response profiles are enriched in nociceptors. (**A**) Example traces from the two drug application protocols used. (**B**) Drug sensitivity and VTD response profiles of 671 neurons from N = 7 mice. (**C**) The frequency of occurrence of VTD profiles in the 511 VTD-responsive neurons shows the same order as in Fig. 2B. The OS profile is the most frequent (50.6 ± 4.2%) then the SD profile (31.2 ± 4.8%) followed by RD (9.4 ± 0.8%) and ID (8 ± 1.8%) (**D**) The proportion of neurons sensitive to at least one agonist (closed bars) and neurons insensitive to any (open bars) within the four VTD response profiles. Larger proportion of SD neurons (71.6 vs. 28.4 ± 2.9%) is insensitive to any of the three agonists. In contrast, Larger proportion of RD neurons (96.7 vs. 3.3 ± 3.3%), ID (85.5 vs. 14.5 ± 7.7%) and OS (81 vs. 19 ± 4.6%) neurons are sensitive to at least one of the three agonists. Percentages were calculated from total number of neurons in each profile (n_(SD)_ = 168, n_(RD)_ = 44, n_(ID)_ = 48, and n_(OS)_ = 251 neuron). Data shown are mean ± SEM. One-way analysis of variance with Sidak’s post-test, ***P < 0.001 and ****P < 0.0001. (**E**) Diameter of SD neurons that are sensitive to at least one of the three agonists (closed bars, mean 21.2 ± 0.9 μm, median 19.6 μm, n = 51 cell) is significantly smaller than that of neurons insensitive to any of the three agonists (open bars; mean 26.9 ± 0.7 μm, median 28.3 μm, n = 117 cell). (**F**) Diameter of VTD-unresponsive neurons sensitive to at least one agonist (closed bars are, mean 20.6 ± 0.4 μm, median 20.7 μm, n = 109 cell) and VTD-unresponsive neurons insensitive to any of the three agonists (open bars, mean 24.4 ± 1 μm, median 24.3 μm, n = 51 cell). In (**E**,**F**), dotted lines represent the mean. Two-tailed unpaired Student’s t-test, *P < 0.05.

Both the SD and RD response profiles were enriched in neurons insensitive to the two nociceptive markers; capsaicin and α, β-methylene ATP. This led us to hypothesise that these two response profiles could be functional markers for non-nociceptors, while the OS and ID response profiles could be functional markers for nociceptors. However, The OS and ID profiles did not occur preferentially in neurons sensitive to the nociceptive markers capsaicin (Fig. 4D) and α, β-methylene ATP (Fig. 5D). Nevertheless, it is possible that the OS neurons insensitive to capsaicin are the same neurons that are sensitive to α, β-methylene ATP and vice versa. To test this hypothesis, it was necessary to apply VTD, capsaicin and α, β-methylene ATP sequentially in the same protocol. We also wanted to profile VTD responses in C-low threshold mechanoreceptors because it was reported that this population is required for injury induced mechanical hypersensitivity^28^. This population expresses TRPA1 but not TRPV1 nor P2×3^29^; therefore we included allyl isothiocyanate (AITC, a specific TRPA1 agonist^30^,^31^,^32^) in our protocol. We applied the five agents in the calcium imaging protocol in two orders, Fig. 6A. The order of agents in the first protocol was; α, β-methylene ATP (1 μM), AITC (100 μM), VTD (30 μM), capsaicin (200 nM) and 40 mM KCl. The order of agents in the second protocol was VTD, ATP, AITC, capsaicin and KCl. As with previous experiments (Figs 4 and 5), there was no significant difference in the proportion of responding neurons nor the frequency of occurrence of VTD profiles in both protocols, Supplementary Fig. S3.

Neurons sensitive to any of the three agonists were three times more likely to respond to VTD (333/442, 75%) than not (109/442, 25%), Fig. 6B. The frequency of occurrence of the four response profiles, Fig. 6C, was similar to previous values in Figs 3C, 4C and 5C. We next examined the 511 VTD-responsive neurons for a relationship between each of the four VTD response profiles and sensitivity to any of the three agonists. The three agonists divided the four VTD profiles into two groups. In one group is the SD response profile which continued to show a bias towards insensitivity to any of the three agonists, Fig. 6D. In the other group are the OS, ID and now the RD response profiles all showing a significant bias towards sensitivity to any of the three agonists, Fig. 6D.

**Figure 7:**
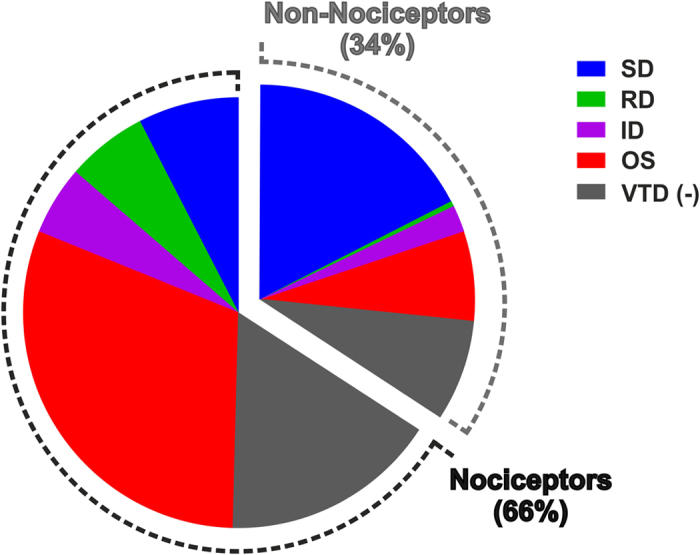
VTD response profiles correlate with classical functional markers of nociceptors. Neurons responding to at least one of the three agonists represent nociceptors and constitute 66% (442/671) of all DRG neurons in culture. Nociceptors with the OS, ID or RD VTD response profiles constitute 64% (282/442) of nociceptors (equivalent 282/671 = 42% of all neurons) compared to 12% (51/442) of nociceptors with the SD profile (equivalent to 51/671 = 8% of all neurons). Neurons insensitive to any of the three agonists represent non-nociceptors and constitute 34% (229/671) of all DRG neurons in culture. Non-nociceptors with the SD VTD response profile constitutes 51% (117/229) of non-nociceptors (equivalent to 117/671 = 17% of all neurons) compared to non-nociceptors with the OS, ID or RD VTD response profiles which constitute 27% (61/229) of non-nociceptors (equivalent 61/671 = 9% of all neurons).

The SD subpopulation of neurons sensitive to any of the three agonists have a smaller mean size (21 μm) compared to the subpopulation insensitive to any of the three agonists (26 μm), Fig. 6E. Interestingly, all neurons unresponsive to VTD but sensitive to any of the three agonists are smaller than 30 μm in diameter, Fig. 6F. The percentage of responsive neurons to 100 μM AITC (54%) is consistent with published findings^31^. The relationship between VTD response profiles and sensitivity to AITC alone matches that for the three agonists combined (Supplementary Fig. S4). From all the above, the OS, ID and RD response profiles are enriched in neurons sensitive to the nociceptive markers whereas the SD response profile is enriched in neurons insensitive to the nociceptive markers, Fig. 7.

**Figure 8:**
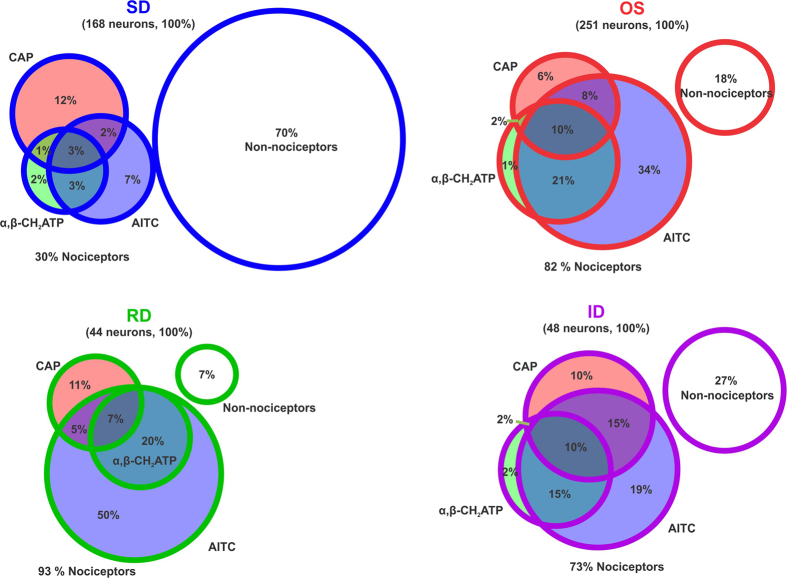
Distribution of sensitivity to the three nociceptive markers within the four VTD response profiles. Venn diagram of the mean percentage of neurons with the SD, OS, RD and ID profiles that responded to allyl isothiocyanate (AITC, blue), capsaicin (CAP, red) and α, β-methylene ATP (α, β-CH_2_ATP, green). The percentage of neurons in each profile that did not respond to any of the agonists comprises the fourth non-overlapping circle (white). Percentages were calculated from the total number of neurons in each VTD profile (SD: 168 cell, OS: 251 cell, RD: 44 cell, and ID: 48 cell) in experiment shown in Fig. 6B.

We also examined the distribution of sensitivity to the three agonists among neurons with the four VTD profiles, Fig. 8. AITC sensitivity was the commonest feature of neurons that responded to one or more of the three agonists with the OS (88%), RD (86%), ID (81%) profiles but not the SD response profile (52%). Neurons with the SD response profile were approximately equally as likely to be capsaicin-sensitive (56%) or AITC-sensitive (52%).

## Discussion

The present study examined VTD-elicited calcium responses in cultured adult mouse DRG neurons. VTD responses were heterogeneous. We categorised VTD responses into four distinct profiles based on the number of peaks and their decay rate. We report that VTD response profiles correlated with soma size and with commonly used pharmacological markers of nociceptor. To our knowledge this is the first detailed characterisation of VTD responses in DRG neurons.

What do VTD response profiles represent? Most of the studies that looked at the effect of VTD on voltage-gated ion channels used patch-clamp electrophysiology to examine the effect of VTD on sodium^8^,^9^, calcium^33^ and potassium^13^ currents separately. In calcium imaging the measured response occurs in non-clamped neurons and is indirect to sodium entry. Therefore, the observed VTD response profiles are the net effect of VTD action on VGSCs at the resting membrane potential and the subsequent activation of other ion channel/exchanger classes. Therefore, it is important to keep in mind that although the response is initiated by VTD’s action on VGSCs, the overall response is shaped by; 1) Qualitative and quantitative differences in the “constellation” of sodium, potassium and calcium voltage-gated ion channels subtypes in each DRG neuron. 2) Variations in calcium buffering mechanisms within each neuron which includes calcium efflux pathways, intracellular stores as well as calcium binding proteins^34^.

Our data shows that 30 μM VTD evoked robust responses in approximately 70% of sensory neurons. A higher dose of 100 μM VTD did not significantly increase the percentage of responding cells, Fig. 1D. Considering that higher doses of VTD are reported to have inhibitory effects on potassium channels, 30 μM seems to be the most suitable concentration to use for an action primarily on VGSCs. VTD predominantly activates TTX-S VGSCs and our results are in agreement with this as TTX blocked most VTD responses (Fig. 1E–G). Voltage-clamp experiments on rat DRG neurons showed that VTD binds TTX-R VGSCs but dissociates at much faster rate than with TTX-S VGSCs^10^, which might explain why the TTX-R VTD responses we observed are transient in nature, Fig. 1F. It remains to be determined which of the TTX-R channels expressed in DRG neurons (i.e. Nav1.8 and Nav1.9) underlie the observed TTX-R VTD responses. We speculate that Nav1.9 is the most likely candidate because its expression is more restricted in DRG than Nav1.8 and this fits with low incidence of TTX-R VTD responses. Secondly, Nav1.9 activation potential is hyperpolarised allowing it to be open at the resting membrane potential which would allow VTD to act on the open channel. In contrast Nav1.8 activation potential is more depolarised and is unlikely to be open at resting membrane potential for VTD to affect it. In fact, it was reported that VTD did not activate a stable cell line expressing Nav1.8^35^. Therefore, the contribution of Nav1.8 to VTD responses will be dependent upon prior action of VTD on TTX-S subtypes to depolarise the membrane. Since this would not happen in the presence of TTX; it is thus unlikely that Nav1.8 produces the TTX-R VTD responses in our experiment.

Importantly, the inability of VTD to activate stable cells expressing Nav1.8 may explain the about 25–30% of DRG neurons that are unresponsive to up to 100 μM VTD, Fig 1D. We suggest that VTD-unresponsive neurons are so because they express mostly TTX-R and little TTX-S channels. In support of this the majority of neurons in this population are less than 30 μm in diameter, Figs 3D, 4F, 5F and 6F. Additionally, the majority of neurons in this population (109/160, 68%) responded to at least one of the three nociceptor markers; capsaicin, AITC and α, β-methylene ATP, Fig. 6B. These 109 neurons constitute 25% of nociceptors (109/442, Fig. 6B). These neurons are likely to represent the small diameter, Nav1.8-rich and high-threshold neurons known as “silent nociceptors”^36^,^37^. Interestingly, silent nociceptors were estimated to be about 30% of all DRG neurons which is about the same percentage as the VTD-unresponsive population. This finding has significant implications for studies using veratridine to activate DRG neurons as it means that up to 25% of nociceptors would not be assayed and their contribution would be unknown.

We categorised VTD calcium responses in cultured DRG neurons into four profiles which we named SD, ID, RD and OS. The four profiles were observed in similar relative frequencies from cultures prepared over many months, from different patches of mice and by different researchers. Furthermore, the frequency of occurrence of VTD profiles was not affected by the order of applications of agonists in our experiments, Supplementary Figs 1–3. The stability of these profiles supports their suitability for use as functional signatures of subpopulations of DRG neurons in drug screens.

The most abundant response type is the OS (47–51% of VTD-responsive neurons) where VTD elicited oscillatory changes in [Ca]i levels. Interestingly, this is the only response profile VTD elicited in N2a and SH-SY5Y cells, Fig. 1B,C. A similar response profile was also reported in bovine chromaffin cells where VTD induced [Ca]i oscillations were dependant on the activation of TTX-S VGSC^25^. The oscillatory response in bovine chromaffin cells were long lasting (up to 40 min) even after VTD wash. A proportion of the OS responses in DRG neurons were persistent but most returned to baseline after VTD wash (not shown). Although SH-SY5Y, N2a and chromaffin cells^12^,^38^,^39^ all express Nav1.7, it is unlikely that the OS profile is a characteristic of Nav1.7 expressing cells as Nav1.7 is expressed in all DRG neurons whereas the OS profile occurs in about 50% of DRG neurons.

The other three types of VTD response profiles shared a profile characterised by a single peak but differed in their decay rate. The difference in the rate of signal decay could be due to a difference in the disassociation rates of VTD from the different VGSC subtypes. In addition, it has been shown that DRG neurons (particularly large-diameter neurons) produce calcium responses with slow decay rates (SD-like responses) when treated with VGKC blockers^40^. Therefore, differences in the decay rates of the VTD response profiles might be in part due to some inhibitory effect of 30 μM VTD on VGKCs. However, this is unlikely to be the main determinant of decay rates as it takes a much higher concentration of VTD (200 μM) to inhibit VGKCs^13^.

**Figure 9:**
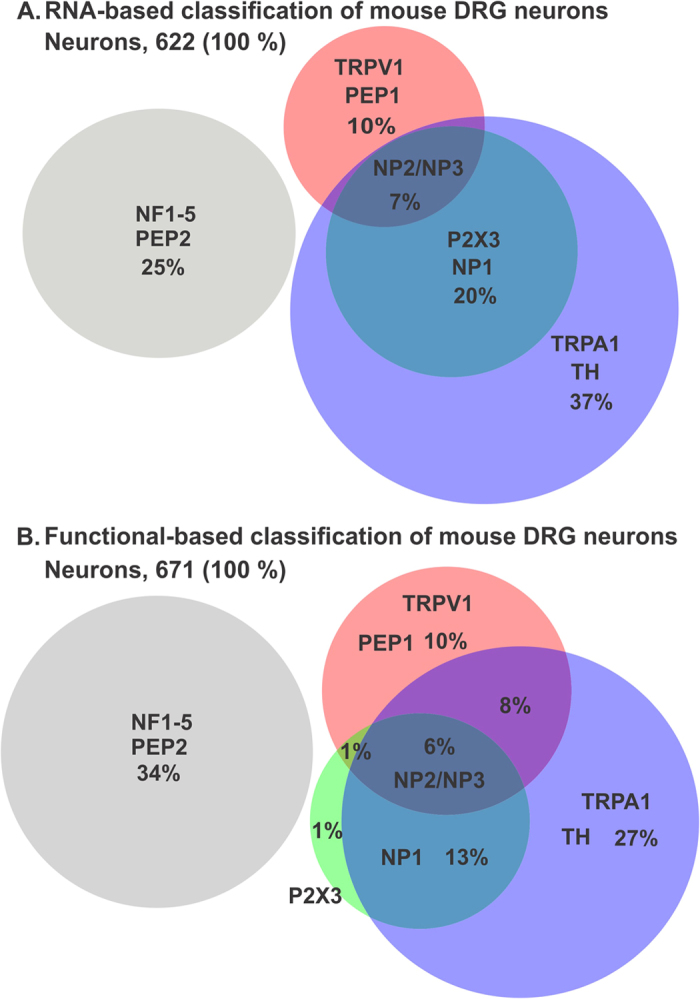
Comparison of the sensitivity to the three nociceptive markers based on published RNASeq and our functional imaging data. (**A**) Venn diagram of the percentage of neurons expressing mRNA for receptors for the three agonists used according to data from Figs 2 and 4 of ref. 29. (**B**) Percentage of neurons responding to the three agonists from our experiment in Fig. 6. The classification of neuronal subpopulations is as per that in ref. 29. NP is non-peptidergic, PEP is peptidergic, NF is neurofilament heavy chain and TH for tyrosine hydroxylase.

A recent paper classified DRG neurons into 11 groups based on their mRNA profile^29^. Using their data (Figs 2 and 4 in ref. 29) we predicted the distribution of sensitivity of DRG neurons to the three agonists used in this study based on the expression of their receptors, Fig. 9A. We compared this to the distribution of sensitivity we obtained from applying the concentrations of agonists we used, Fig. 9B. The two distributions have many similarities. For example, all non-peptidergic neurons (represented by groups NP1–3 in ref. 29) are predicted to respond to AITC and in agreement we found that almost all α, β-methylene -ATP responded to 100 μM AITC. Another similarity is that the TRPA1 positive population is the largest of the three agonists being 64% of DRG neurons by mRNA expression of TRPA1 and 54% by response to AITC. The percentage of AITC responders (54%) is almost identical to that reported by Barabas, M.E. *et al*.^31^ (55%). Therefore, the 10% higher percentage of TRPA1 expressing neurons must be due to the higher sensitivity of mRNA detection compared to functional imaging. The same reason is likely to explain the 9% higher percentage of neurons not responding to any of the agonists in our hands compared to what is predicted by mRNA expression. Capsaicin activated 25% DRG neurons in our experiments as opposed to a predicted 17% based on expression of TRPV1 receptor. The percentage of capsaicin responders in our experiments is in agreement with others (e.g.ref. 22). The higher percentage in our and others’ experiments could be due increased TRPV1 expression in cultured DRG neurons (>1 day) compared to disassociated but non-cultured neurons as used for the mRNA sequencing study.

The data presented here raises several interesting questions. One question is what are the key molecular determinants of each of the four VTD response profiles? Answering this allows the use of VTD profiles as readout for these ion channels and exchangers in high throughput functional screens on primary DRG neurons. Considering that 25–30% of nociceptors do not respond to 30 μM VTD and that these are likely to be “silent” nociceptors, a second question is does the sensitization of DRG neurons by inflammation and nerve injury affect the number of VTD responsive neurons and their response profiles? Answering this question would allow the use of VTD response profiles as readout of drugs’ ability to cause or reverse sensitisation of DRG neurons in high throughput screens. Such screens have the advantage of showing the net effect of a drug on the various classes of ion channels that give rise to VTD response profiles.

In conclusion, the present study shows that VTD induces calcium responses with heterogeneous profiles in TTX-S rich sensory neurons. VTD response profiles reflect distinct subpopulations of sensory neurons. These subpopulations overlap but are not identical to the subpopulations identified by classical functional nociceptive markers, Fig. 7. The OS and RD profiles are particularly enriched in nociceptors (neurons sensitive to at least one of the three agonists), while the SD profile is enriched in non-nociceptors (neurons insensitive to any of the three agonists). Our findings provide a detailed characterisation of VTD action on the different subsets of DRG neurons. Our work is relevant to studies and screens using VTD to activate DRG neurons.

## Methods

### DRG culture

Adult male C57BL/6 mice were sacrificed according to Schedule 1 of the Animal (Scientific procedure) Act 1986. DRG from all spinal levels were isolated and collected in PBS. PBS was then replaced with 1 mL Dulbecco’s Modified Eagle’s Medium/F12 (DMEM/F12) with Glutamax medium (Gibco) containing Dispase (1 mg/mL, Sigma) and Collagenase Type XI (0.6 mg/mL, Sigma) and left for 60 min at 37 °C and 5% CO_2_. DRGs were then triturated with a P1000 pipette tip. The cell suspension was carefully layered on top of 15% Bovine Serum Albumin (Melford) in DMEM/F12 and centrifuged at 800 g, for 10 min at room temperature with the minimum deceleration speed. The cell pellet was then washed in culture medium composed of DMEM/F12 plus 10% Fetal Bovine Serum (FBS, Gibco), 100 units/ml penicillin and 100 μg/ml streptomycin (Gibco). Cells were pelleted again, re-suspended in DRG culture medium and plated on glass coverslips coated with polyornithine (20 μg/mL, Sigma). Cells were imaged 24 h after plating.

### Calcium imaging

DRG neurons were loaded with 2 μM Fura-2, AM (Molecular Probes) in standard extracellular solution (140 mM NaCl, 4 mM KCl, 2 mM CaCl_2_, 10 mM HEPES, 5 mM glucose, pH = 7.4 with NaOH) and incubated for 30 min at 37 °C. Coverslips were then washed with standard extracellular ringer solution and left for 15 min at 37 °C and 5% CO_2_ and then for another 15 min at room temperature. Cells were excited with 350 and 380 nm for ratiometric measurement of intracellular calcium using Cairn Dual OptoLED system. Cells were viewed using a 40X oil immersion objective. Images were acquired using a Hamamatsu C4742–95 camera. The cells were perfused with standard extracellular solution for at least 5 min to establish stable baseline. All recordings were performed at room temperature (23 ± 1 °C). Drugs were perfused at a flow rate of 3 mL/min. High potassium extracellular solution (104 mM NaCl, 40 mM KCl, 2 mM CaCl_2_, 10 mM HEPES, 5 mM glucose, pH = 7.4 with NaOH) was perfused at the end of the recordings, unless mentioned otherwise, as a depolarizing agent to identify viable neurons. *Simple PCI 6* software was used for data acquisition, background subtraction and Fura-2,AM ratiometric measurement (F350/380 nm).

### Compounds

All drugs were made to the required working dilution in standard extracellular ringer solution from stock solutions of the following concentrations: VTD (5 mM in ethanol, Abcam ab120279), Capsaicin (10 mM in ethanol, Tocris 0462), α, β-methylene ATP (10 mM in water, Sigma M6517), TTX (30 μM in citrate buffer, Abcam Asc-054), TNP-ATP triethylammonium salt (10 mM in water, Tocris 2464), and allyl isothiocyanate (AITC; 100 μM, Sigma 377430).

### Data and statistical analysis

Neurons were identified by their responsiveness to 40 mM KCl. We defined a response as an increase in (F350/380) ratio of > 6 SD above the baseline. Differences in fluorescence (ΔF/F_0_) were calculated according to the following formula: F350/380 ratio in the presence of drug (F) during drug application – the mean of F350/380 ratio of the 2.5 min prior to drug application (F_0_). Statistical analysis was performed by calculating the mean percentage of responsive neurons (n) from the indicated number of mice for each figure (N). Sample mean of each set of experiments was calculated from multiple independent experiments (as specified in the result section) and compared to each other by one-way analysis of variance (ANOVA) with Sidaks’ post-test. Cell diameter measurements were performed using ImageJ software; cell area was obtained from a hand-drawn line delineating the soma. Soma diameter was then calculated using the following formula, Diameter = √(4.Area/π). For cell size comparison, two-tailed unpaired Student’s t-test was used for comparisons. All statistical analysis and comparisons were performed by *GraphPad Prism* software (version 7.00 for Windows). Area-proportional Venn diagrams were generated by *BioVenn* software^41^ and colour edited by *CorelDRAW X8* software.

## Additional Information

**How to cite this article:** Mohammed, Z. A. *et al*. Veratridine produces distinct calcium response profiles in mouse Dorsal Root Ganglia neurons. *Sci. Rep.* **7**, 45221; doi: 10.1038/srep45221 (2017).

**Publisher's note:** Springer Nature remains neutral with regard to jurisdictional claims in published maps and institutional affiliations.

## Supplementary Material

Supplementary Figures

## References

[b1] Wood J. N. . Ion channel activities implicated in pathological pain. Novartis Foundation symposium 261, 32–40, discussion 40–54 (2004). 15469043

[b2] Lai J., Porreca F., Hunter J. C. & Gold M. S. Voltage-gated sodium channels and hyperalgesia. Annual review of pharmacology and toxicology 44, 371–397, doi: 10.1146/annurev.pharmtox.44.101802.121627 (2004). 14744251

[b3] Waxman S. G. The molecular pathophysiology of pain: abnormal expression of sodium channel genes and its contributions to hyperexcitability of primary sensory neurons. Pain Suppl 6, S133–140 (1999). 10491982 10.1016/S0304-3959(99)00147-5

[b4] Felix J. P. . Functional assay of voltage-gated sodium channels using membrane potential-sensitive dyes. Assay and drug development technologies 2, 260–268, doi: 10.1089/1540658041410696 (2004). 15285907

[b5] Cao Z. . Influence of lipid-soluble gating modifier toxins on sodium influx in neocortical neurons. The Journal of pharmacology and experimental therapeutics 326, 604–613, doi: 10.1124/jpet.108.138230 (2008). 18448863 PMC2852114

[b6] Stevens M., Peigneur S. & Tytgat J. Neurotoxins and their binding areas on voltage-gated sodium channels. Frontiers in pharmacology 2, 71, doi: 10.3389/fphar.2011.00071 (2011). 22084632 PMC3210964

[b7] Ulbricht W. Effects of veratridine on sodium currents and fluxes. Reviews of physiology, biochemistry and pharmacology 133, 1–54 (1998). 10.1007/BFb0000612 9600010

[b8] Ulbricht W. The effect of veratridine on excitable membranes of nerve and muscle. Ergebnisse der Physiologie, biologischen Chemie und experimentellen Pharmakologie 61, 18–71 (1969). 10.1007/BFb0111446 4903416

[b9] Barnes S. & Hille B. Veratridine modifies open sodium channels. The Journal of general physiology 91, 421–443 (1988). 2454286 10.1085/jgp.91.3.421 PMC2216135

[b10] Farrag K. J., Bhattacharjee A. & Docherty R. J. A comparison of the effects of veratridine on tetrodotoxin-sensitive and tetrodotoxin-resistant sodium channels in isolated rat dorsal root ganglion neurons. Pflugers Archiv: European journal of physiology 455, 929–938, doi: 10.1007/s00424-007-0365-5 (2008). 17962978

[b11] Augusti-Tocco G. Neuroblastoma culture: an experimental system for the study of cellular differentiation. Trends in Biochemical Sciences 1, 151–154, doi: 10.1016/0968-0004(76)90416-3.

[b12] Biedler J. L., Roffler-Tarlov S., Schachner M. & Freedman L. S. Multiple neurotransmitter synthesis by human neuroblastoma cell lines and clones. Cancer research 38, 3751–3757 (1978). 29704

[b13] Verheugen J. A., Oortgiesen M. & Vijverberg H. P. Veratridine blocks voltage-gated potassium current in human T lymphocytes and in mouse neuroblastoma cells. The Journal of membrane biology 137, 205–214 (1994). 8182730 10.1007/BF00232589

[b14] Sigel E. Effects of veratridine on single neuronal sodium channels expressed in Xenopus oocytes. Pflugers Archiv: European journal of physiology 410, 112–120 (1987). 2446243 10.1007/BF00581903

[b15] Teichert R. W. . Characterization of two neuronal subclasses through constellation pharmacology. Proceedings of the National Academy of Sciences of the United States of America 109, 12758–12763, doi: 10.1073/pnas.1209759109 (2012). 22778416 PMC3411979

[b16] Snider W. D. & McMahon S. B. Tackling pain at the source: new ideas about nociceptors. Neuron 20, 629–632 (1998). 9581756 10.1016/s0896-6273(00)81003-x

[b17] Lawson S. N., Caddy K. W. & Biscoe T. J. Development of rat dorsal root ganglion neurones. Studies of cell birthdays and changes in mean cell diameter. Cell and tissue research 153, 399–413 (1974). 4458950 10.1007/BF00229167

[b18] Rambourg A., Clermont Y. & Beaudet A. Ultrastructural features of six types of neurons in rat dorsal root ganglia. Journal of neurocytology 12, 47–66 (1983). 6842273 10.1007/BF01148087

[b19] Lawson S. N. Phenotype and function of somatic primary afferent nociceptive neurones with C-, Adelta- or Aalpha/beta-fibres. Experimental physiology 87, 239–244 (2002). 11856969

[b20] Harper A. A. & Lawson S. N. Conduction velocity is related to morphological cell type in rat dorsal root ganglion neurones. J Physiol 359, 31–46 (1985). 3999040 10.1113/jphysiol.1985.sp015573 PMC1193363

[b21] Cavanaugh D. J. . Restriction of transient receptor potential vanilloid-1 to the peptidergic subset of primary afferent neurons follows its developmental downregulation in nonpeptidergic neurons. The Journal of neuroscience: the official journal of the Society for Neuroscience 31, 10119–10127, doi: 10.1523/jneurosci.1299-11.2011 (2011). 21752988 PMC3147010

[b22] Ren A. J. . ZBTB20 regulates nociception and pain sensation by modulating TRP channel expression in nociceptive sensory neurons. Nature communications 5, 4984, doi: 10.1038/ncomms5984 (2014). PMC6687506 25369838

[b23] Chen C. C. . A P2X purinoceptor expressed by a subset of sensory neurons. Nature 377, 428–431, doi: 10.1038/377428a0 (1995). 7566119

[b24] Collo G. . Cloning OF P2X5 and P2X6 receptors and the distribution and properties of an extended family of ATP-gated ion channels. The Journal of neuroscience: the official journal of the Society for Neuroscience 16, 2495–2507 (1996). 8786426 10.1523/JNEUROSCI.16-08-02495.1996 PMC6578782

[b25] Lewis C. . Coexpression of P2X2 and P2X3 receptor subunits can account for ATP-gated currents in sensory neurons. Nature 377, 432–435, doi: 10.1038/377432a0 (1995). 7566120

[b26] Bradbury E. J., Burnstock G. & McMahon S. B. The expression of P2X3 purinoreceptors in sensory neurons: effects of axotomy and glial-derived neurotrophic factor. Molecular and cellular neurosciences 12, 256–268, doi: 10.1006/mcne.1998.0719 (1998). 9828090

[b27] Virginio C., Robertson G., Surprenant A. & North R. A. Trinitrophenyl-substituted nucleotides are potent antagonists selective for P2X1, P2X3, and heteromeric P2X2/3 receptors. Molecular pharmacology 53, 969–973 (1998). 9614197

[b28] Seal R. P. . Injury-induced mechanical hypersensitivity requires C-low threshold mechanoreceptors. Nature 462, 651–655, doi: 10.1038/nature08505 (2009). 19915548 PMC2810205

[b29] Usoskin D. . Unbiased classification of sensory neuron types by large-scale single-cell RNA sequencing. Nature Neurosicence 18, 145–153, doi: 10.1038/nn.3881 (2015). 25420068

[b30] Jordt S. E. . Mustard oils and cannabinoids excite sensory nerve fibres through the TRP channel ANKTM1. Nature 427, 260–265, doi: 10.1038/nature02282 (2004). 14712238

[b31] Barabas M. E., Kossyreva E. A. & Stucky C. L. TRPA1 is functionally expressed primarily by IB4-binding, non-peptidergic mouse and rat sensory neurons. PloS one 7, e47988, doi: 10.1371/journal.pone.0047988 (2012). 23133534 PMC3485059

[b32] Paulsen C. E., Armache J. P., Gao Y., Cheng Y. & Julius D. Structure of the TRPA1 ion channel suggests regulatory mechanisms. Nature 520, 511–517, doi: 10.1038/nature14367 (2015). 25855297 PMC4409540

[b33] Romey G. & Lazdunski M. Lipid-soluble toxins thought to be specific for Na+ channels block Ca2+ channels in neuronal cells. Nature 297, 79–78 (1982). 6280075 10.1038/297079a0

[b34] Lu S. G., Zhang X. & Gold M. S. Intracellular calcium regulation among subpopulations of rat dorsal root ganglion neurons. J Physiol 577, 169–190, doi: 10.1113/jphysiol.2006.116418 (2006). 16945973 PMC2000672

[b35] Liu C. J. . A high-capacity membrane potential FRET-based assay for NaV1.8 channels. Assay and drug development technologies 4, 37–48, doi: 10.1089/adt.2006.4.37 (2006). 16506887

[b36] Djouhri L. . The TTX-resistant sodium channel Nav1.8 (SNS/PN3): expression and correlation with membrane properties in rat nociceptive primary afferent neurons. J Physiol 550, 739–752, doi: 10.1113/jphysiol.2003.042127 (2003). 12794175 PMC2343087

[b37] Schmidt R. . Novel classes of responsive and unresponsive C nociceptors in human skin. The Journal of neuroscience: the official journal of the Society for Neuroscience 15, 333–341 (1995). 7823139 10.1523/JNEUROSCI.15-01-00333.1995 PMC6578337

[b38] Lou J. Y. . Fibroblast growth factor 14 is an intracellular modulator of voltage-gated sodium channels. J Physiol 569, 179–193, doi: 10.1113/jphysiol.2005.097220 (2005). 16166153 PMC1464207

[b39] Wada A., Yanagita T., Yokoo H. & Kobayashi H. Regulation of cell surface expression of voltage-dependent Nav1.7 sodium channels: mRNA stability and posttranscriptional control in adrenal chromaffin cells. Frontiers in bioscience: a journal and virtual library 9, 1954–1966 (2004). 14977601 10.2741/1314

[b40] Teichert R. W. . Functional profiling of neurons through cellular neuropharmacology. Proceedings of the National Academy of Sciences of the United States of America 109, 1388–1395, doi: 10.1073/pnas.1118833109 (2012). 22307590 PMC3277115

[b41] Hulsen T., de Vlieg J. & Alkema W. BioVenn – a web application for the comparison and visualization of biological lists using area-proportional Venn diagrams. BMC Genomics 9, 1–6, doi: 10.1186/1471-2164-9-488 (2008). 18925949 PMC2584113

[b42] Onizuka S. . Capsaicin indirectly suppresses voltage-gated Na+ currents through TRPV1 in rat dorsal root ganglion neurons. Anesthesia and analgesia 112, 703–709, doi: 10.1213/ANE.0b013e318204ea5b (2011). 21156986

