# Supplementary material for: Veratridine produces distinct calcium response profiles in mouse Dorsal Root Ganglia neurons
Source: Sci Rep. 2017 Mar 24;7:45221. doi: 10.1038/srep45221 (PMC5364547; doi:10.1038/srep45221)
Supplement: Supplementary Figures [file srep45221-s1.pdf]

Veratridine produces distinct calcium response profiles in mouse Dorsal Root Ganglia neurons

Zainab A Mohammed, Ciara Doran, David Grundy and Mohammed A Nassar*

Biomedical Science, University of Sheffield, Sheffield S102TN, UK

*Corresponding Author m.nassar@sheffield.ac.uk

Supplementary figures to Zainab et al

Figure S1:

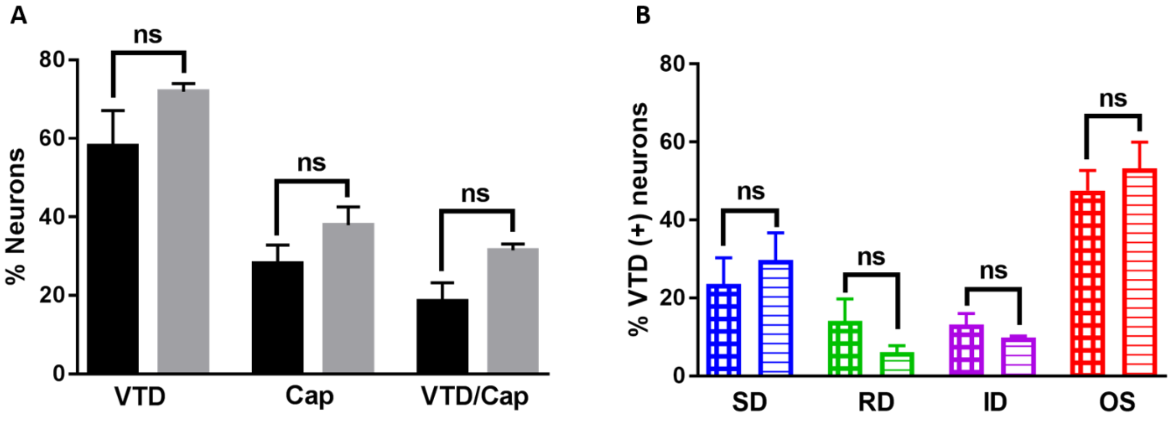

Supplementary S1: Order of veratridine and capsaicin application does not affect VTD response profiles. A. Percentage of neurons that responded to VTD, capsaicin (CAP) and VTD plus capsaicin (VTD+/CAP+) when VTD was applied before 1 µM capsaicin (black bars; N = 5 mice, n = 200 cell) and when VTD was applied after 200 nM capsaicin (grey bars; N = 3 mice, n = 379 cell). Percent responses between VTD first and VTD second protocols were not significantly different. Percent of neurons responsive to VTD (58 + 9 %, 112 cell vs. 71.8 + 2 %, n = 274 cell); percent of neurons sensitive to capsaicin (28.1 + 5 %, n = 59 cell vs. 37.9 + 4.6 %, n = 164 cell) and percent of neurons responsive to both VTD and capsaicin (31.5 + 1.6 %, n = 119 cell vs. 18.5 + 5 %, n = 37 cell) were not different. **B.** The frequency of occurrence of the four VTD profiles is not different when VTD is applied first (crossed bars, N = 3 mice) and second (horizontal lines, N= 3 mice). The frequencies for SD response profile are (23.6 + 7, n = 29 cell vs. 29.7 + 7 %, n = 82 cell), for RD response profile are (14.1 + 6 %, n = 13 cell vs. 6.3 + 2 %, n = 17 cell), for ID response profile are (13.2 + 3 %, n = 15 cell vs. 9.8 + 0.4 %, n = 27 cell) and for OS response profile are (47.5 + 5 %, n = 53 cell vs. 53.3 + 7 %, n = 145 cell). Data shown are mean + SEM. One-way analysis of variance with Sidak's post-test.

Figure S2:

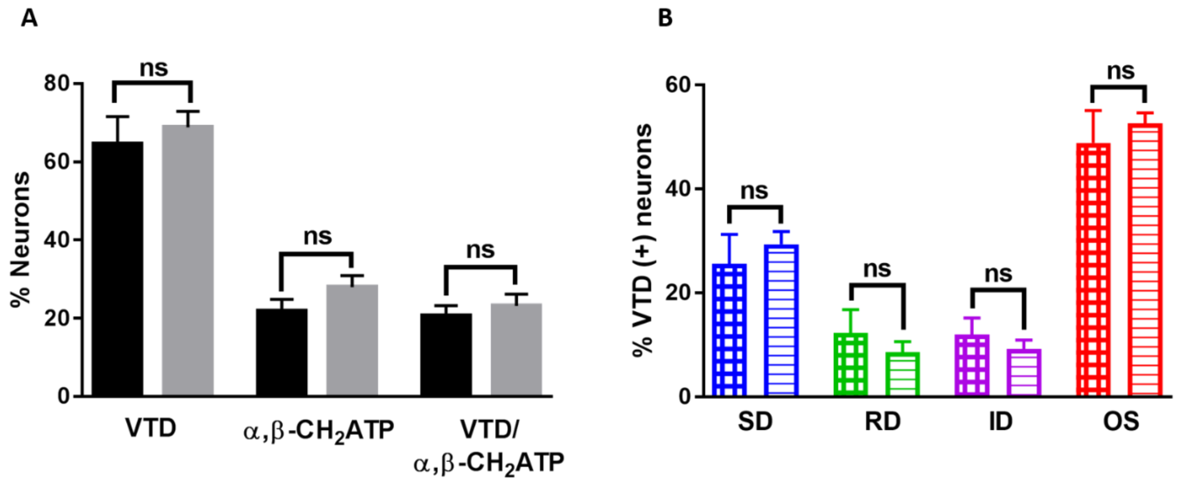

Supplementary S2: the order of veratridine and α, β-methylene ATP application does not affect VTD response profiles. A. Percentage of neurons that responded to VTD, α, β-methylene ATP (α, β-CH_2ATP) and VTD plus α, β-methylene ATP (VTD/α, β-CH_2ATP) when VTD was applied before α, β-methylene ATP (black bars; N = 3 mice, n = 294 cell) and when VTD was applied after α, β-methylene ATP (grey bars; N = 6 mice, n = 1051 cell). Percent responses between VTD first and VTD second protocols were not significantly different. Percent of neurons responsive to VTD (64.7 ± 7, n = 201 cell vs. 68.8 ± 4 %, n = 711 cell); percent of neurons sensitive to α, β-methylene ATP (21.8 ± 3 %, n = 69 cell vs. 27.9 ± 3 %, n = 275 cell); and percent of neurons sensitive to both VTD and capsaicin (20.7 ± 3 %, n = 65 cell vs. 23.2 ± 3 %, n = 224 cell) are not different. **B.** The frequency of occurrence of the four VTD profiles is not different when VTD is applied first (crossed lines, N = 3 mice) and second (horizontal lines, N= 6 mice). The frequencies for the SD response profile are (25.6 ± 6, n = 50 cell vs. 29.4 ± 3 %, n = 208 cell), for the RD response profile are (12.3 ± 5 %, n = 20 cell vs. 8.6 ± 2%, n = 62 cell), for the ID response profile are (12 ± 3%, n = 20 cell vs. 9.2 ± 2 %, n = 70 cell) and for the OS response profile are (48.9 ± 6 %, n = 108 cell vs. 52.7 ± 2 %, n = 370 cell). Data shown are mean ± SEM. One-way analysis of variance with Sidak's post-test.

Figure S3:

A

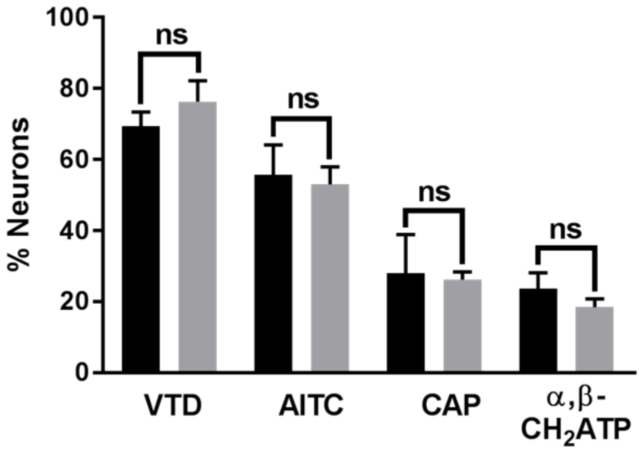

B

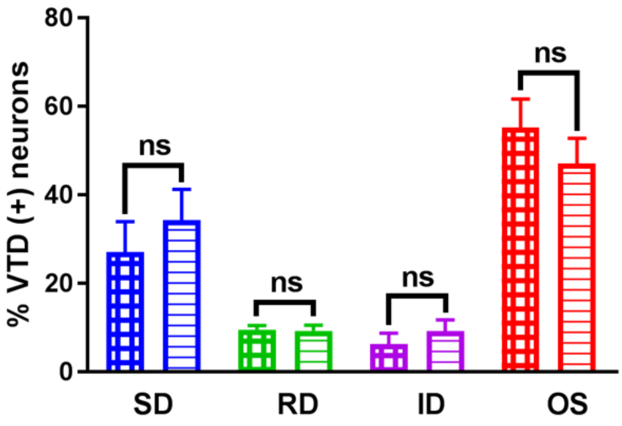

Supplementary S3: The order of Veratridine, α, β-methylene ATP, capsaicin and AITC application does not affect VTD response profiles. A. Percentage of neurons responding to VTD, allyisothiocyanate (AITC), capsaicin (CAP), and α, β-methylene ATP (α, β-CH_2ATP) when VTD was applied first (black bars; N = 3 mice, n = 220 cell) and when VTD was applied third (grey bars; N = 4 mice, n = 451 cell). Percent responses between VTD first and VTD second protocols were not significantly different. Percent of neurons responsive to VTD (69.4 ± 4 %, n = 150 cell vs. 76.3 ± 6 %, n = 361 cell), AITC (55.7 ± 8.5 %, n = 128 cell, vs. 53.1 ± 5 %, n = 229 cell), CAP (28 ± 11 %, n = 58 cell vs. 26.2 ± 2.1 %, n = 115), and α, β-methylene ATP (23.7 ± 4.4 %, n = 54 cell vs. 18.5 ± 2.3 %, n = 87 cell) are not different. **B.** The frequency of occurrence of the four VTD profiles is not different when VTD is applied first (crossed bars, N = 3 mice) and third (horizontal lines, N= 4 mice). The frequencies for the SD response profile are (27.1 ± 6.9 %, n = 13 cell vs. 34.3 ± 7 %, n = 38 cell), for the RD response profile are (9.5 ± 1 %, n = 15 cell vs. 9.3 ± 1.3 %, n = 26 cell), for the ID response profile are (6.3 ± 2.5 %, n = 9 cell vs. 9.3 ± 2.5 %, n = 26 cell) and for the OS response profile are (55.2 ± 6.5 %, n = 71 cell vs. 47.2 ± 5.6 %, n = 135 cell). Data shown are mean ± SEM. One-way analysis of variance with Sidak's post-test.

Figure S4:

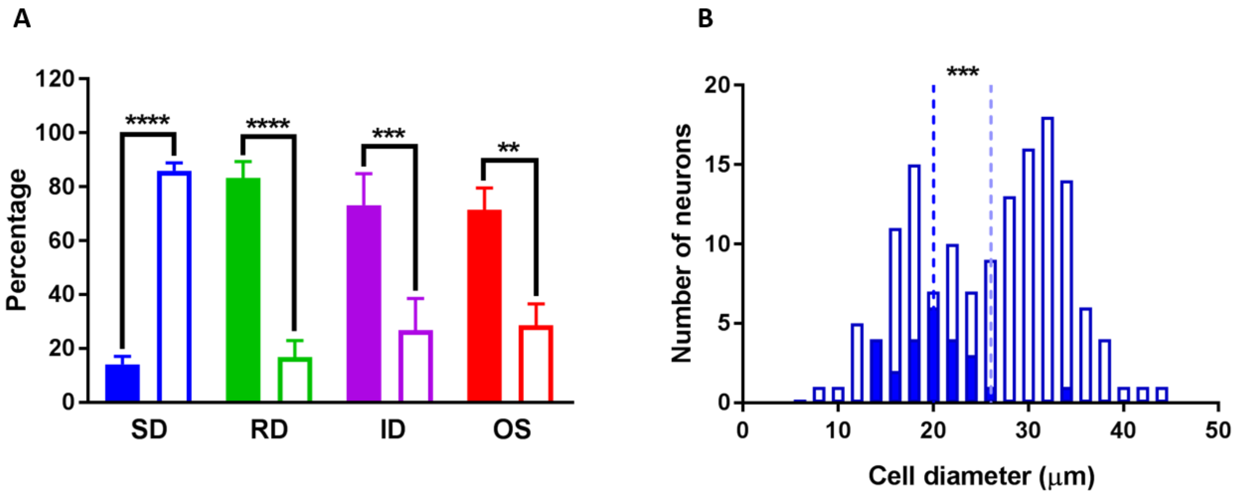

Supplementary S4: Relationship between AITC sensitivity and the four veratridine profiles. A. This data is extracted from data presented in figure 6. The proportion of AITC-sensitive (closed bars) and AITC-insensitive neurons (open bars) within each of the four VTD response profiles. Significantly a larger proportion of neurons with the SD (86 vs. 14 + 3 %) profile is insensitive to AITC. In contrast, significantly a larger proportion of neurons with the RD (83.2 vs. 16.8 + 6.2 %), ID (73.2 vs. 16.8 + 11.7 %) and OS (71.5 vs. 28.5 + 8 %) profiles are sensitive to AITC. Percentages were calculated from total number of neurons in each profile ($n_{(SD)}$ = 168, $n_{(RD)}$ = 44, $n_{(ID)}$ = 48, and $n_{(OS)}$ = 251 neuron). Data shown are mean $\underline{+}$ SEM. One-way analysis of variance with Sidak's post-test, **P < 0.01, **P < 0.001 and ****P < 0.0001. **B.** Histogram of diameter of neurons with the SD response profile showing that neurons sensitive to AITC (closed bars; mean 20 + 0.9 µm, median 19.5 µm, n = 25 cell) are significantly smaller than neurons insensitive to AITC (open bars; mean 26.1 + 0.6 µm, median 27.9 µm, n= 143 cell). Dotted lines indicate the mean. Two-tailed unpaired Student's t-test, *P < 0.05.
